# Neuroendocrine Differentiation in Prostate Cancer: A Mechanism of Radioresistance and Treatment Failure

**DOI:** 10.3389/fonc.2015.00090

**Published:** 2015-04-14

**Authors:** Chang-Deng Hu, Richard Choo, Jiaoti Huang

**Affiliations:** ^1^Department of Medicinal Chemistry and Molecular Pharmacology, Purdue University Center for Cancer Research, Purdue University, West Lafayette, IN, USA; ^2^Department of Radiation Oncology, Mayo Clinic, Rochester, MN, USA; ^3^Department of Pathology, David Geffen School of Medicine at UCLA, Los Angeles, CA, USA

**Keywords:** neuroendocrine differentiation, prostate cancer, CREB, ATF2, radiosensitization, radiotherapy, cancer stem cell, EMT

## Abstract

Neuroendocrine differentiation (NED) in prostate cancer is a well-recognized phenotypic change by which prostate cancer cells transdifferentiate into neuroendocrine-like (NE-like) cells. NE-like cells lack the expression of androgen receptor and prostate specific antigen, and are resistant to treatments. In addition, NE-like cells secrete peptide hormones and growth factors to support the growth of surrounding tumor cells in a paracrine manner. Accumulated evidence has suggested that NED is associated with disease progression and poor prognosis. The importance of NED in prostate cancer progression and therapeutic response is further supported by the fact that therapeutic agents, including androgen-deprivation therapy, chemotherapeutic agents, and radiotherapy, also induce NED. We will review the work supporting the overall hypothesis that therapy-induced NED is a mechanism of resistance to treatments, as well as discuss the relationship between therapy-induced NED and therapy-induced senescence, epithelial-to-mesenchymal transition, and cancer stem cells. Furthermore, we will use radiation-induced NED as a model to explore several NED-based targeting strategies for development of novel therapeutics. Finally, we propose future studies that will specifically address therapy-induced NED in the hope that a better treatment regimen for prostate cancer can be developed.

## Introduction

Prostate cancer is the second leading cause of cancer death among men in developed countries (1). In 2015, it is estimated that 27,540 men will die from prostate cancer in US according to American Cancer Society. Most of these deaths are due to the progression of localized diseases into metastatic, castration-resistant, prostate cancer (CRPC).

Based on prostate specific antigen (PSA) level, tumor grade, and the extent of primary tumor in the prostate gland, clinically localized prostate cancer is classified into low-risk (PSA ≤10 ng/ml, Gleason score ≤6, and stage T1c–T2a), intermediate-risk (PSA >10 but ≤20 ng/ml, Gleason score 7, or stage T2b), and high-risk (PSA >20, Gleason score ≥8, or stage T2c) (2, 3). While a majority of low-risk disease is cured with surgery or radiotherapy (RT), intermediate- and high-risk disease has a relatively high rate of recurrence following a definitive therapy. For example, approximately 30–50% of high-risk, clinically localized, prostate cancer treated with RT develop a biochemical recurrence within 5 years post-therapy, and about 20% die of prostate cancer within 10 years (4–7). Given that about 25% of patients are diagnosed with a high-risk disease at presentation (8), there has been a major effort to develop a strategy to optimally manage this group of patients in recent years.

Resistance to RT (radioresistance) can be intrinsic or acquired (9). Given the heterogeneity of prostate cancer cells, it is likely that certain cells have intrinsic radioresistance, whereas others have the ability to acquire radioresistance over the course of RT. This review discusses the recent advance in our understanding of radiation-induced neuroendocrine differentiation (NED) and the implication on RT efficacy, and proposes possible approaches to addressing radiation-induced NED.

## Neuroendocrine Differentiation as a Mechanism of Therapy Resistance

Normal prostate tissue consists of three types of epithelial cells: basal cells, luminal cells, and neuroendocrine (NE) cells. Unlike basal cells and luminal cells, NE cells constitute only <1% of total epithelial cells, and their physiological role remains unclear (10). In prostate adenocarcinoma, the presence of an increased number of neuroendocrine-like (NE-like) cells is observed (10–14). It has been hypothesized that these NE-like cells may arise from luminal-type prostate cancer cells by a NED or transdifferentiation process (15–17). NE-like cells do not proliferate, and lack the expression of androgen receptor (AR) and PSA.

**Figure 1:**
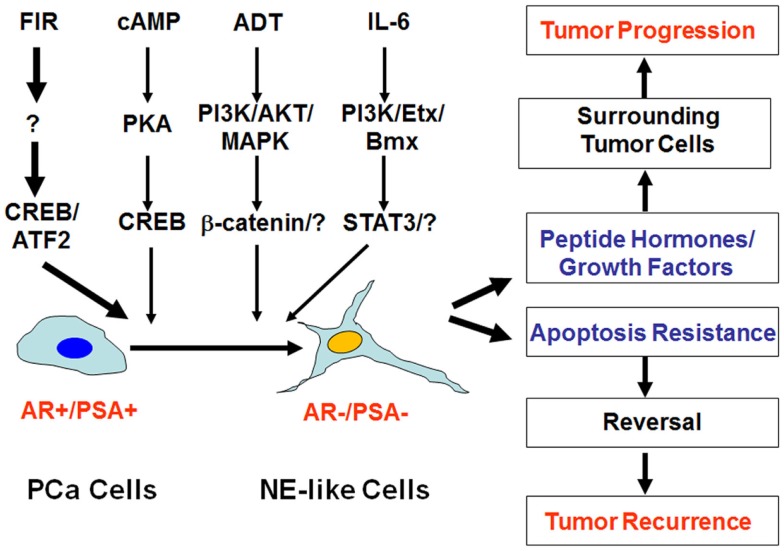
**Impact of neuroendocrine differentiation on prostate cancer progression and tumor recurrence**. Neuroendocrine differentiation (NED) can be induced by fractionated ionizing radiation (FIR), cAMP, androgen-deprivation therapy (ADT), and IL-6 via distinct signaling pathways. The clinical impact of NED on prostate cancer progression and therapy response can be twofold. On the one hand, NE-like cells can produce peptide hormones and growth factors to promote tumor progression. On the other hand, the dormant and apoptosis-resistant NE-like cells may resume the ability to proliferate due to the reversibility of NED, and contribute to treatment failure and tumor recurrence.

Clinical observations have suggested that NED correlates with disease progression and poor prognosis (14, 16, 18–28). Several mechanisms may account for the impact of NED on prostate cancer progression and therapeutic responses. First, NE-like cells do not proliferate, and thus they function as a dormant phenotype making NE-like cells particularly resistant to therapies. Second, NE-like cells express high levels of survival genes such as survivin and Bcl-2 (29–31), or exhibit alteration in calcium homeostasis (32), again conferring resistance to treatments. Third, NE-like cells secrete a number of peptide hormones and growth factors to support the growth of surrounding tumor cells in a paracrine manner. Lastly, NED is a reversible process (33, 34). For example, treatment of LNCaP cells with cAMP or cAMP-inducing agents induces NED within a few days (33). Interestingly, removal of cAMP or cAMP-inducing agents results in either retraction or shedding of the neuritic processes within 10 h. Also, within 2 days the expression of neuron specific enolsase (NSE), a biomarker of NE and NE-like cells, returns to basal levels. Similarly, NED induced by androgen depletion (e.g., charcoal-stripped fetal bovine serum-containing medium) can be reversed by culturing cells in normal serum-containing medium. Based on these observations, there are two possible pathways by which NED can contribute to disease progression and therapy failure (Figure 1). One is that NE-like cells can survive therapeutic interventions and thus contribute to tumor recurrence if they resume proliferation post treatments. Second, the presence of NE-like cells supports the growth of surrounding tumor cells in a paracrine manner, thus conferring to disease progression.

## Pre-Existing NED Versus Therapy-Induced NED in Prostate Cancer

### Pre-existing NED

Although NE-like cells in adenocarcinoma share many characteristics of normal NE cells, they also differ in some aspects. For example, NE-like cells express some luminal cell markers, whereas NE cells express some basal cell markers (15). Accumulating evidence favors the hypothesis that NE-like cells come from a transdifferentiation process of prostate cancer cells, either from hormone-naïve or CRPC (15). There are numerous stimuli and agents, which likely activate distinct signaling pathways to induce NED (15, 35). For example, cAMP signaling may activate the PKA/CREB signaling pathway to induce NED (33, 36–41), whereas IL-6-induced NED appears to be mediated by activation of the PI3K/Etk/Bmx and STAT3 pathways (35, 36, 42–46) (Figure 1). Interestingly, while EGF may prevent androgen depletion-induced NED in an MAPK and PI3K/AKT-dependent manner (47), it may also promote NED in LNCaP cells in an ErbB2-dependnt manner if treated with an inhibitor of the PI3K/AKT pathway such as LY294002 (48, 49). Because activation of the cAMP signaling pathway and the PI3K/AKT pathways are often associated with prostate cancer development and progression, it is very likely that a subset of cells may undergo NED during prostate cancer development and progression. Thus, these NE-like cells are already present at the time of initial diagnosis of prostate cancer, and this pre-existing NED confers resistance to subsequent treatments such as RT, androgen-deprivation therapy (ADT), and chemotherapy (14, 16, 18–28).

### Therapy-induced NED

Therapy-induced NED refers to acquired NED induced by a therapeutic agent. Such therapeutic agents include ADT (50–52) and docetaxel (23, 53). Recently, it has been shown that enzalutamide and abiraterone (two recently FDA-approved agents for the treatment of CRPC) can also induce NED and that induced NED is correlated with poor survival in CRPC patients (54, 55). Consistent with these clinical observations, induction of NED in prostate cancer cells by androgen depletion is well established *in vitro* (34, 47, 56–59) and in prostate cancer xenografts in mice (59–64). Lin et al. recently reported that a patient-derived xenograft line showed a complete induction of NED following castration (compared to no sign of NED prior to castration) (65). These observations provide convincing evidence that castration does induce NED.

### RT can also induce NED

While working on the isolation of radiation-resistant sublines after a fractionated RT regimen (2 Gy/day, 5 days/week), we unexpectedly found the display of apparent neurite outgrowth by irradiated cells after a 4-week fractionated ionizing radiation (FIR) (66). Immunoblotting analysis confirmed that these cells express high levels of NE markers chromogranin A (CgA) and NSE, indicating that FIR also induces NED *in vitro*. Furthermore, it was observed that FIR-induced LNCaP xenograft tumors to undergo NED in nude mice, which displayed a four to fivefold increase of serum CgA after 4-week FIR (67). Consistent with this observation, in a pilot clinical study, we measured serum CgA in nine patients who were treated with RT, and found that four out of nine patients showed 1.5- to 2.2-fold increase in serum CgA after 7-week RT (67). Similarly, Lileby et al. also found that a subset of prostate cancer patients treated with RT showed elevated serum CgA levels 3 months after the treatment (21). However, these pilot clinical studies have neither addressed the issue of whether RT-induced CgA elevation correlates with RT failure nor have they established the relationship between the disease status and the extent of serum CgA elevation. Nevertheless, it is clear that NED can be induced by clinical therapeutic agents including RT (acquired NED), and therapy-induced NED may represent one of the mechanisms leading to treatment failure.

## The Relationship between NE-Like Cells, Cancer Stem Cells, Senescent Cells, and Epithelial-Mesenchymal Transition

Based on the expression of marker proteins in NE cells, luminal cells, and basal cells, it was suggested that NE-like cells arise from prostate cancer cells by a process of NED or transdifferentiation (15). However, there is also evidence suggesting that NE-like cells are derived from neural crest cells or stem cells as extensively reviewed by Conteduca et al. (17). Palapattu et al. examined the expression of cancer stem cell marker CD44 in LNCaP, DU-145, and PC-3 cells (68), and revealed that CD44 is only expressed in cells that are positive for NE markers. Consistent with this observation, the correlation between CD44 expression and NE markers (NSE and CgA) was also observed in prostate cancer tissues. Interestingly, 100% of prostatic small cell NE carcinomas, an aggressive variant of prostate cancer that is composed of highly proliferating NE cells, have CD44 expression, whereas its expression was detectable only in a minority of small cell NE carcinoma from other organs. This observation raised an interesting possibility that CD44 expression may be a useful biomarker to distinguish the origin of prostate small cell NE carcinoma from NE carcinoma in other organs. Because CD44 positive cells are capable of generating CD44 negative cells, are highly tumorigenic, and express several “stemness” genes (69), these findings support the hypothesis that CD44 positive NE-like cells are prostate cancer cell stem cells.

Recently, Kyjacova et al. used clinically relevant FIR to irradiate four human prostate cancer cell lines, and observed that there are two populations of survived cells: one is adherent, senescent-like cells, and the other is non-adherent, anoikis-resistant stem cell-like cells (70). However, since the authors did not examine the expression of NE markers, it remains unknown whether one or both populations also express NE markers. We previously isolated several sublines from irradiated LNCaP cells that lost the expression of CgA and NSE (66). All three sublines could not be induced to undergo NED by FIR. Because NED, cancer stem cells, and epithelial–mesenchymal transition (EMT) share similar properties (17), it would be interesting to examine whether these sublines exhibit properties of cancer stem cells, senescent cells, and/or mesenchymal cells. Nonetheless, these observations suggest that FIR treatment may selectively enrich the population of cancer stem cells or induce NED, senescence, and/or EMT. Several mechanisms may account for this. First, NE-like cells, cancer stem cells, and EMT or senescent cells may have the same origin (e.g., stem cells); thus, the type of phenotypic changes may depend on the type of stimuli. Second, NED, cancer stem cells, and EMT or senescence may have a significant overlap of signaling molecules that are required for the development and maintenance of each of these phenotypic changes (17). For example, expression of Snail, a major transcription factor implicated in the induction of EMT, also induces NED in LNCaP cells (71). Third, these phenotypic changes share common inducers, which could lead to induction of NED, stemness, EMT, or senescence. In fact, stress signaling, such as hypoxia, can induce both NED (72) and EMT (73), as well as enrich the cancer stem cell subpopulation (74). Finally, considering cell heterogeneity, the cellular populations may consist of all of these cell types that are induced by distinct stimuli. Future cell lineage analysis and single cell analysis will likely provide insight into the origin of NE-like cells and their relationship with other cell types.

## Mechanism of Radiation-Induced NED

**Figure 2:**
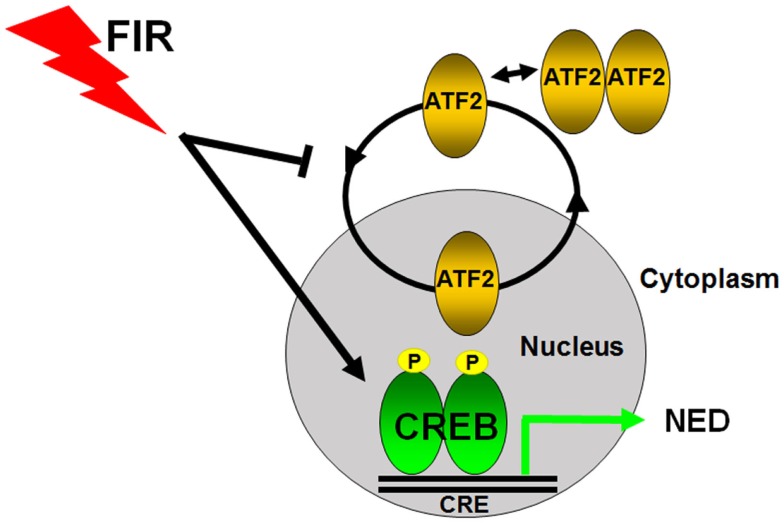
**Transcriptional regulation of radiation-induced neuroendocrine differentiation in prostate cancer cells**. CREB and ATF2 belong to the same CREB/ATF family transcription factors to regulate gene transcription by binding to the same cAMP response element (CRE). ATF2 constantly shuttles as a monomric form between the nucleus and cytoplasm in prostate cancer cells, and its nuclear regulation is tightly regulated. CREB acts as a transcriptional activator and ATF2 functions as a transcriptional repressor of NED in prostate cancer cells. Fractionated ionizing radiation (FIR) induces NED by activating CREB and impairing the nuclear import of ATF2.

To study how NED is regulated at the transcriptional level, we examined the subcellular localization of ATF2 and observed increased cytoplasmic localization (66). ATF2 is a member of activator protein 1 (AP-1) family of proteins (75, 76). We discovered that ATF2 is a nucleocytoplasmic shuttling protein that possesses two nuclear import motifs and two nuclear export motifs (77, 78). ATF2 shuttles in LNCaP cells and IR impairs its nuclear import (66). Given that ATF2 belongs to the ATF/CREB family, and CREB is known to both regulate CgA transcription (79) and act downstream of the cAMP signaling (20, 80), we examined the expression and activation of CREB, and found that IR activated CREB as well as increased nuclear localization of phosphorylated CREB at Ser133 (66). These results suggest that CREB is a transcriptional activator of NED while ATF2 is a transcriptional repressor of NED, and that FIR tilts the balance between CREB and ATF2, leading to cell differentiation (Figure 2). Indeed, expression of a constitutively activated CREB is sufficient to induce NED, whereas expression of a constitutively nuclear-localized ATF2 (nATF2) can antagonize CREB-induced NED (66). Consistent with the converse roles of CREB and ATF2, nATF2, or a non-phosphorylatable CREB (CREB133A) also inhibits FIR-induced NED. Likewise, we recently established stable cell lines expressing several CREB short hairpin RNAs (shRNAs), and found that CREB knockdown significantly inhibited FIR-induced neurite outgrowth and NSE expression (81). However, CgA expression was not inhibited which was surprising given that CREB can activate CgA transcription. Because the CREB family members form different homodimers or heterodimers, the inability of CREB knockdown to inhibit CgA expression may be explained by functional compensation of other dimeric complexes. To overcome this, we established another stable cell line that has inducible expression of ACREB, a dominant negative CREB in which the basic region is replaced by acidic amino acids hence deficient in DNA-binding. This ACREB forms a dimeric complex not only with CREB but also with other CREB family members, exhibiting a potent inhibitory effect on the expression of CREB target genes (82, 83). Indeed, ACREB expression increased radiation-induced cell death by more than 70% in the setting of 40 Gy FIR treatment. Importantly, expression of ACREB both during the first 2 weeks (acquisition of radioresistance) and during the second 2 weeks (acquisition of NED phase) increased FIR-induced cell death (81). This result not only demonstrates the critical role of CREB in FIR-induced NED but also provides evidence that targeting either phase could be an effective approach to developing novel radiosensitizers.

## Multiple Phases of Radiation-Induced NED

**Figure 3:**
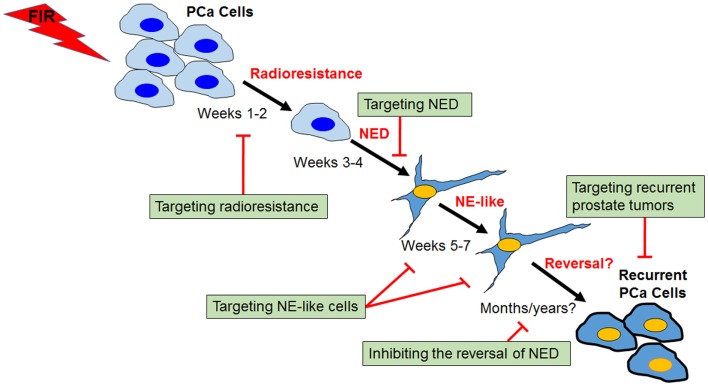
**Process and targeting strategies of radiation-induced neuroendocrine differentiation in prostate cancer cells**. Shown is a schematic view of several distinct phases of fractionated ionizing radiation (FIR)-induced NED in prostate cancer cells (PCa). The critical role of CREB in the acquisition of radioresistance and NED phases has been demonstrated, and identification of upstream regulators of CREB may lead to development of novel radiosensitizers. Targeting NE-like cells and inhibiting the reversal of the “dormant” NE-like cells to a proliferating state could also be clinically useful. Further, profiling radioresistant recurrent prostate cancer cells may allow identification of molecules contributing to cross-resistance of recurrent prostate cancer after radiotherapy failure, and ultimately may lead to the development of novel therapeutic agents for the treatment of recurrent prostate tumors.

Fractionated ionizing radiation-induced NED differs from androgen depletion- and cAMP-induced NED in that cancer cells must survive from the treatment first. Unlike cAMP- and androgen depletion-induced NED in which almost all LNCaP cells can be induced to differentiate into NE-like cells, we observed that cell growth was largely inhibited during the first week of irradiation, and increased cell death became apparent during the second week of irradiation. However, little cell death was observed starting from the third week onward. Instead, cells began to show neurite outgrowth and cell body became smaller. With continued irradiation, cells showed extended neurite outgrowth (66). Upon 4 weeks of irradiation, almost all survived cells differentiated into NE-like cells and continued irradiation for another 3 weeks did not induce cell death. Similar processes were observed in DU-145 and PC-3 cells, though the extent of NED appears to be less than LNCaP cells (67). These observations suggest that FIR-induced NED constitutes several distinct phases: acquisition of radioresistance during the first 2 weeks, acquisition of NED during the second 2 weeks, maintenance of NED during the last 3 weeks, and reversal to the proliferating state after the completion of the FIR treatment (Figure 3).

## Strategies Targeting Radiation-Induced NED

A number of approaches have been attempted to target NE-like cells by either blocking secreted neuropeptide-mediated effects or inhibiting the survival signaling pathways in NE-like cells (17). However, the clinical effect of these therapeutic maneuvers remains unclear. Because NED can be induced by a variety of stimuli and therapeutic agents, the underlying molecular mechanisms of NED need to be thoroughly investigated so that targeted therapies can be developed accordingly. This is particularly important for therapy-induced NED. Further, recurrent tumors derived from therapy-induced NE-like cells may behave differently. For example, RT- and chemotherapy-induced NED involves a clonal selection, and likely reprograming of survival cells. These cells are likely cross-resistant to other treatments (66).

Using radiation-induced NED as a model, we hypothesize here that two complementary directions could be pursued to develop novel therapeutics. One is to identify targets and pathways that are specific for the acquisition of radioresistance and NED, and the other is to identify molecules that are critical for the maintenance of NE-like phenotype. In addition, developments of agents that inhibit the reversal of NE-like cells or target recurrent tumors after RT failure should also be considered.

### Targeting acquisition of radioresistance and differentiation phases

Because NED can be induced by a variety of stimuli via activation of distinct mechanisms, targeting specific signaling pathways downstream of a particular inducer is a reasonable strategy. Application of such targeting agents (applied as either a single agent or a combination of multiple agents) would therefore inhibit therapy-induced NED. In the case of RT-induced NED, we have demonstrated that the CREB signaling is critical for FIR-induced NED (66, 67). To determine whether targeting RT-induced NED can be explored to develop a novel radiosensitizer, we established doxycycline-inducible expression system to diminish CREB activity by expressing either ACREB, a dominant negative mutant of CREB, or shRNAs to knockdown CREB. The availability of these two inducible CREB targeting approaches allowed us to specifically test whether targeting CREB during the first 2 weeks or during the second 2 weeks can sensitize prostate cancer cells to radiation. Our results showed that targeting CREB during either phase can increase FIR-induced cell death (81). This finding not only confirms that CREB is critical for FIR-induced NED but also suggests that targeting FIR-induced NED can sensitize prostate cancer cells to radiation. Since several CREB targeting agents are being developed (84), it would be interesting to test whether these agents are effective in inhibiting FIR-induced NED. Furthermore, identification of upstream regulators, e.g., protein kinases, could provide an important approach to targeting FIR-induced NED. In conclusion, this type of targeting agents can be developed as radiosensitizers by targeting either the acquisition of radioresistance, NED phase, or both phases.

### Targeting NE-like cells

Because NE-like cells do not proliferate and rather stay as “dormant” cells, cytotoxic chemotherapeutic agents may not be effective. It is therefore necessary to understand how these “dormant” cells survive and maintain their phenotype. It is possible that an autocrine pathway confers cell survival and would be a potential target for therapeutics. Alternatively, we may target the survival pathway. For example, NE-like cells often overexpress survivin (29), and several survivin-targeting agents have been developed (85). It would be interesting to determine if targeting survivin can induce apoptosis of therapy-induced NE-like cells.

### Inhibiting the reversal of NE-like cells

One of the potential impact of NED on tumor recurrence is its reversibility. Like cAMP- and androgen depletion-induced NED (33, 34, 58), FIR-induced NED may also be reversible (66). The molecular mechanisms underlying this process remain unclear. However, inhibiting the reversal of NE-like cells to a proliferating state may be clinically useful if the reversibility of NE-like cells does occur in prostate cancer patients.

### Targeting recurrent prostate cancer cells

Treatment of recurrent prostate cancer remains a major challenge. A therapy for recurrent tumor is variable, and depends on the type of primary treatment. For example, a treatment strategy for recurrent prostate cancer after RT failure is different from that for recurrent prostate cancer after surgery. This is because recurrent prostate cancer after RT has undergone genetic and epigenetic changes under the selective pressures, and may be cross-resistant to other treatments. Consistent with this notion, isolated radioresistant sublines after 40 Gy of FIR are indeed cross-resistant to androgen depletion and docetaxel (66). Given that 30–50% of high-risk and 10% of low-risk prostate cancer recur after RT, it is urgently needed to develop agents that can specifically target recurrent prostate cancer after RT failure. Because the recurrent tumor is composed of heterogeneous cells, including NE-like cells or cancer stem cells as discussed above, comparative analysis of genetic and epigenetic changes as well as signaling pathways between multiple radioresistant sublines and parental cells may lead to identification of molecular alterations that are common to all recurrent cells. If identified, molecular alterations could be validated with recurrent prostate cancer specimens, and developing novel therapeutics targeting specifically for RT-failed recurrent prostate cancer may become possible.

## Future Perspectives

### Animal models to study the impact of NED in prostate cancer progression and therapeutic response

The impact of NED on prostate cancer progression has been well demonstrated *in vivo*. It was shown that the implantation of NE mouse prostate allograft (NE-10) in nude mice bearing LNCaP xenograft tumors on the opposite flank can support the growth of LNCaP xenograft tumors under castration condition (86). This study provides compelling evidence that factors secreted by NE tumors are sufficient to support the growth of prostate tumors under castration condition (86). Consistent with this, Deeble et al. elegantly demonstrated again in castrated condition that coinjection of the constitutively activated protein kinase A subunit-induced NE-like cells and LNCaP cells into nude mice enhanced tumor growth (38). These studies corroborated *in vitro* findings that conditioned medium from NE-like culture can stimulate the growth of prostate cancer cells (38, 87), and that secreted mitogenic neuropeptides such as neurotensin are critical for the stimulation of tumor cell growth (33, 36, 87). Interestingly, Valerie et al. also showed that treating prostate cancer cells expressing high levels of neurotensin receptor 1 (NTR1) with a selective NTR1 antagonist SR48692 sensitizes prostate cancer cells to ionizing radiation. Thus, secreted neurotensin from NE-like cells not only promotes prostate cancer cell growth but also confers the surrounding tumor cells radioresistance. Although these studies provide evidence that secreted neuropeptides and growth factors from NE-like cells *in vivo* can promote prostate cancer progression and alter therapeutic responses, these findings are limited to established cell lines in immunocompromised mice and thus further research must be done with a better model system.

While many genetically engineered mouse (GEM) models have been established to study the development, progression, and therapeutic responses of prostate cancer (88), a GEM model that allows for the elucidation of the impact of NED on prostate cancer progression and therapeutic response is unavailable. By transgenically overexpressing SV40 large T antigen, a TRAMP mouse model was established, which has a high incidence of NE tumor arising from prostate with a high potential to metastasize to lung, liver, and other tissues (89). The TRAMP mouse model is more representative of human NE carcinoma, a rare type of prostate cancer present at initial clinical presentation or in some ADT-treated setting (88). Recently, Qi et al. found that knockout of Siah2, a ubiquitin ligase, completely suppresses the development of NE tumors in the background of TRAMP (90), demonstrating a critical role of this E3 ligase in the development of NE tumors. Molecular analysis further revealed that HIF-1α, which is stabilized by Siah2, mediates the effect of Siah2 to selectively regulate, in combination with FoxA2, the expression of HIF target genes that are required for or involved in the development of NE tumor. Although these studies provide genetic evidence that Siah2, HIF-1α, and FoxA2 are required for the regulation of NE tumor development at the transcription level, the TRAMP mouse model does not permit the analysis of the impact of pre-existing and therapy-induced focal NED on disease progression and therapeutic response. Given that castration-induced NED also occurs in other GEM models (91, 92), it would be interesting to test if FIR also induces NED in these GEM models. Further, innovative approaches (e.g., inducible NED mouse models, chemical probes) that allow manipulation of NE-like cells or NED in these GEM models will likely facilitate the study of NED impact on prostate cancer progression and radiation response. As castration-induced NED has also been reported in patient-derived xenograft model system (65), infecting the cells with lentiviruses (that can inducibly destroy NE-like cells during the course of FIR treatment) will similarly permit the study of acquired NED in radio-responsiveness.

### Clinical diagnosis of NED in prostate cancer patients

Traditionally, the proteins such as CgA, NSE, synaptophin, and others that are expressed by NE-like and NE cells are used as biomarkers to identify NE-like or NE cells in tissue specimens using immunohistochemistry. However, analysis is often confounded by various factors including a sampling issue, leading to conflicting outcomes. Thus, it is generally felt that immunohistochemical analysis may not accurately represent the status of NED in a given patient. To overcome this, serum biomarkers have been used and their correlation to NED in tissues have been examined. It was found that CgA is the best biomarker to reflect NED in tissue (93). To date, serum CgA has been used to monitor ADT-induced NED and chemotherapy-induced NED (23, 24, 27, 53–55, 94, 95). We and others have also observed serum CgA elevation in some patients who were treated with RT (21, 67). Because prostate cancer cells express a basal level of CgA, and activation of transcription factors (e.g., CREB) may also lead to increased synthesis of CgA, measurement of individual biomarkers may not accurately reflect the status of NED in tissues. In addition, obtaining a biopsy for the examination of NED in cancer tissues in post-RT setting is very challenging. Thus, it is very desirable to develop new methods that can reliably diagnose NED in cancer tissues. One approach is to test whether circulating tumor cells can be used to monitor NED in patients in addition to serum CgA measurement. Alternatively, measurement of multiple biomarkers may be necessary for a more accurate diagnosis. One example is the ratio of CgA/PSA. Measurement of serum CgA in irradiated xenograft tumors revealed that the ratio of serum CgA/PSA might provide a better prediction of NED (67). Given that NE-like cells are PSA-low or negative and can secrete CgA, future research should focus on their relationship and the correlation with clinical outcomes.

### Potential impact of current treatment modalities on radiotherapy-induced NED

#### Evaluation of current treatment modalities for locally advanced diseases

Locally advanced, high-risk, prostate cancer currently poses therapeutic challenges. Currently, the standard management for this group of patients is a combined treatment of RT plus ADT. The rationale for combining RT with ADT was based on the fact that both treatments can kill cancer cells or suppress cancer cell growth, and that the combination may lead to a synergistic effect. Indeed, several phase III clinical studies have demonstrated that RT plus ADT provides a survival benefit, in comparison with either RT or ADT alone (4, 96–99). The rationale for adding ADT in the RT setting is that ADT can eliminate androgen-dependent clones, potentiate the tumoricidal effect of RT, and may eradicate micrometastatic disease (96). However, whether ADT can radiosensitize prostate cancer cells is unknown. In fact, *in vitro* studies using LNCaP cells suggest that androgen depletion did not radiosensitze LNCaP cells in clonogenic assays, though apparent additive effect was observed (100). Given that ADT induces NED in a subpopulation of cancer cells (50–52), it would be necessary to evaluate the impact of this combined therapeutic approach on therapy-induced NED, in comparison to a monotherapy setting (ADT or RT alone). Ideally, developing novel therapeutic agents that not only sensitize prostate cancer cells to RT but also inhibit therapy-induced NED would be ideal and likely initiate a paradigm shift for future management of prostate cancer.

#### Impact of new treatment modalities on RT-induced NED

Radiotherapy is one of the main curative modalities for localized prostate cancer. Advances have been made to improve the efficacy of RT in recent years. These include a dose-escalation strategy, a hypofractionation regimen, an incorporation of chemotherapy, and a new RT modality such as high-dose-rate brachytherapy and proton therapy (101–108). Although biological, physical, and clinical rationales clearly support the use of these treatment modalities, their impact on radiation-induced NED remains unstudied. It is worth mentioning that all nine patients enrolled in our pilot clinical study were treated with proton therapy (67). As such, it could be critical to compare the effect of various other RT protocols or modalities on radiation-induced NED. Because FIR-induced NED is completed by a 4-week of irradiation, a dose-escalation strategy over a protracted course likely has a minimal effect on radiation-induced NED. However, other treatment strategies such as an ultra-hypofractionation regimen (e.g., five treatments over 1–2 weeks) or high-dose-rate brachytherapy (given over 1–2 weeks) may have less extent of radiation-induced NED. Also, proton therapy may have less degree of radiation-induced NED, as it has a higher relative biological effectiveness in comparison to a conventional photon beam. The decrease in radiation-induced NED may, in turn, translate to a clinical benefit with improved treatment outcomes. On a translational research perspective, it would be worthwhile to determine whether the observed clinical benefit correlates with the extent of radiation-induced NED. If so, this would provide a biological rationale for exploring different RT regimens or modalities aiming to minimize radiation-induced NED and may also allow for reduction or possible elimination of the use of adjuvant ADT in RT setting.

## Conclusion

Although NED has been a well-recognized phenotypic change in prostate cancer, its impact on prostate cancer progression and therapeutic responses has only recently gained significant attention. Several studies have provided compelling evidence that pre-existing NED confers resistance to treatments such as RT. However, the impact of therapy-induced NED on disease progression and treatment failures has not been rigorously studied. Using FIR-induced NED as a model system, we have provided evidence that targeting FIR-induced NED is an effective radiosensitizing approach. Future research should be directed at understanding the molecular mechanisms by which FIR induces NED and confers acquired radioresistance as well as tumor recurrence. With the use of appropriate animal models, implementation of new technologies as well as methodologies to diagnose RT-induced NED and better understanding of the biological effect of novel treatment modalities, we hope that a better RT strategy will be developed and implemented in clinical practice in the future.

## Conflict of Interest Statement

The authors declare that the research was conducted in the absence of any commercial or financial relationships that could be construed as a potential conflict of interest.
